# Herring oil rich in long-chain monounsaturated fatty acid (C22: 1n-11) lowers plasma lipids and modulates fatty acid composition, oxidation, and inflammation in rats

**DOI:** 10.3389/fnut.2025.1611166

**Published:** 2025-09-26

**Authors:** Camilla H. Nundal, Siri Lunde Tungland, Hege G. Bakke, Pavol Bohov, Thomas A. Aloysius, Arild C. Rustan, Bodil Bjørndal, Aurora Brønstad, Jannike Øyen, Suzanne Brandt, Magne O. Sydnes, Ottar Nygård, Simon N. Dankel, Lise Madsen, Rolf Kristian Berge

**Affiliations:** ^1^Department of Safety, Chemistry and Biomedical Laboratory Sciences, Western Norway University of Applied Sciences, Bergen, Norway; ^2^Department of Chemistry, Bioscience and Environmental Engineering, University of Stavanger, Stavanger, Norway; ^3^Section for Pharmacology and Pharmaceutical Biosciences, Department of Pharmacy, University of Oslo, Oslo, Norway; ^4^Mito Biotech AS, Bergen, Norway; ^5^Department of Sport, Food, and Natural Sciences, Western Norway University of Applied Sciences, Bergen, Norway; ^6^Department of Clinical Medicine, University of Bergen, Bergen, Norway; ^7^Institute of Marine Research, Bergen, Norway; ^8^Department of Chemistry, University of Bergen, Bergen, Norway; ^9^Department of Heart Disease, Haukeland University Hospital, Bergen, Norway; ^10^Department of Clinical Science, University of Bergen, Bergen, Norway

**Keywords:** fatty acid, herring oil, lipid metabolism, mitochondria, peroxisome

## Abstract

**Introduction:**

Marine oils and fatty fish rich in long-chain n-3 polyunsaturated fatty acids (PUFAs), such as eicosapentaenoic acid (C20:5n-3, EPA) and docosahexaenoic acid (C22:6n-3, DHA), have been reported to enhance fatty acid (FA) oxidation and reduce plasma triacylglycerol and cholesterol levels. In addition to n-3 PUFAs, herring oil contains long-chain monounsaturated fatty acids (MUFAs), including cetoleic acid (C22:1n-11). This study aimed to investigate the effect of consuming CETO3^®^ oil—derived from herring and naturally rich in n-3 PUFAs and cetoleic acid—on plasma lipid levels, FA composition, mitochondrial oxidation, and inflammation in rats.

**Methods:**

Rats were fed low-fat diets supplemented with 5% CETO3^®^ oil (experimental) or soy oil (control) for 10 weeks. Plasma lipid profile (triglycerides (TG), total cholesterol, low-density lipoprotein (LDL) and high-density lipoprotein (HDL)) and FA composition in both liver and plasma were analyzed. *In vitro* substrate oxidation was assessed using ¹⁴CO₂-trapping in human liver and human myotubes. Safety parameters, including blood hematology, glucose tolerance, and organ weights, were also measured.

**Results:**

CETO3^®^ supplementation decreased plasma levels of total fat (−58%), TG (−55%), total cholesterol (−41%), and LDL cholesterol (−45%), while increasing the ratio of HDL to LDL cholesterol (46%). Supplementation also increased hepatic and plasma levels of long-chain n-3, n-9, and n-11 MUFAs, including C22:1n-11, and decreased n-6 FA accumulation. The reduction in saturated long-chain FAs in both the liver and plasma indicated increased hepatic peroxisomal and mitochondrial activity. Furthermore, increased oleic acid oxidation was observed in human myotubes in the presence of C20:1n-11 and C20:1n-9.

**Discussion:**

These findings suggest that intake of CETO3^®^ oil lowers plasma lipids, potentially through enhanced peroxisomal and mitochondrial FA oxidation. The shift in FA composition, with reduced n-6 FAs and increased n-3 and n-11 MUFAs, indicates an anti-inflammatory effect. CETO3^®^ oil also appears safe, as hematological parameters, glucose tolerance, and organ weights remained unaffected.

## 1 Introduction

The incidence of cardiometabolic syndrome is rapidly increasing, contributing to a rise in cardiovascular diseases (CVDs). Numerous CVDs are linked to atherosclerosis, characterized by a progressive accumulation of lipid-rich plaques within arterial walls. Elevated blood lipid levels, particularly low-density lipoprotein (LDL) cholesterol, play a central role in the initiation and progression of atherosclerosis, ultimately increasing the risk of events such as myocardial infarction (1–3). Despite the increased use of cholesterol-lowering therapies, atherosclerosis remains the leading cause of death worldwide, accounting for approximately 18 million deaths annually. Although elevated cholesterol levels are a major risk factor, growing evidence suggests that inflammation also plays a crucial role in the initiation, progression, and complications of the disease (4–6). Therefore, the mortality associated with CVDs underscores the importance of prevention and the need to identify strategies to reduce their incidence.

Consumption of fatty fish and fish oil has been linked to a lower incidence of sudden cardiac death and a decrease in the total mortality rate across several studies (7, 8). Fish oils are rich in long-chain n-3 polyunsaturated fatty acids (PUFAs), such as eicosapentaenoic acid (C20:5n-3; EPA) and docosahexaenoic acid (C22:6n-3; DHA). The therapeutic potential of n-3 PUFAs has been extensively studied across a wide range of conditions, including CVDs. Potential mechanisms through which n-3 PUFAs may reduce the risk of CVD include their beneficial effects on lipid and lipoprotein metabolism and inflammatory responses (9–11). Marine-origin lipids also contain varying amounts of unique fatty acids (FAs). For example, herring, as well as marine mammals such as seals and whales, are rich in long-chain monounsaturated fatty acids (MUFAs), which are derived from their food sources, such as zooplankton (12–15). The major long-chain MUFAs in marine sources include gadoleic acid (C20:1n-11) and cetoleic acid (C22:1n-11) from the n-11 series. Additionally, gondoic acid (C20:1n-9) and erucic acid (C22:1n-9) from the n-9 series are also present in certain vegetable oils, such as those derived from mustard seeds and rapeseed (16).

Although numerous reports and reviews in recent years have demonstrated beneficial effects of marine-derived n-3 PUFAs on CVDs and lipid metabolism, few studies have focused on the effects of long-chain MUFAs. The purpose of this investigation was to evaluate whether the consumption of CETO3^®^, an oil derived from herring that is rich in cetoleic acid, EPA, and DHA, affects lipid metabolism in the liver and plasma, as well as its effects on cardiometabolic risk factors. In addition, we sought to determine whether mitochondrial FA oxidation contributes to FA mobilization.

## 2 Materials and methods

### 2.1 Animal study and diets

Animal studies were approved by the Norwegian Animal Research Authority (license number FOTS ID 30111). Male Wistar rats (*Rattus Norvegicus*), 5 weeks old, were purchased from Taconic (Denmark). Upon arrival, they were randomized and acclimatized for 1 week with unrestricted access to chow and water, under 12 h light/dark cycles at 22 ± 2 °C and 55 ± 5% humidity. At the start of the experiments, the rats were block-randomized to their respective interventions, with eight rats per group. The rats were fed low-fat diets providing 16% of total energy from fat (lard), 64% from carbohydrates, and 20% from protein. To these diets, 5% (w/w) of either CETO3^®^ (Grøntvedt Biotech AS, Norway) for the experimental group or soy oil for the control group was added, resulting in final diets with approximately 20% of energy from fat, 61% from carbohydrates, and 19% from protein. Feed was provided in fixed amounts, with leftovers weighed after 10 weeks, and weight gain was measured once weekly. A relatively high dietary inclusion level was chosen based on previous studies reporting inconsistent effects of monounsaturated fatty acids on blood lipid profiles. The oils were incorporated into the diets as a fixed proportion (5%) rather than as weight-adjusted doses (e.g., mg/kg body weight), following the feeding strategy established for this experiment. This approach may have resulted in some variations in individual intake due to differences in actual food consumption among animals. All animals survived the experiment. The rats were anesthetized with 2–5% isoflurane and decapitated, and blood was collected into EDTA tubes and centrifuged, with plasma stored at −80 °C. The organs were collected, weighed, snap-frozen, and stored at −80 °C.

### 2.2 Laboratory analyses

Blood screening was performed at termination. Biochemical lipid analyses in rat fasting plasma were performed at Haukeland University Hospital using the Cobas 8,000 system (Roche Diagnostics), following standard laboratory protocols to measure triglycerides (TG), total cholesterol, LDL cholesterol, and high-density lipoprotein (HDL) cholesterol. Non-HDL cholesterol was calculated by subtracting HDL from total cholesterol. For hematology parameters, whole blood samples were analyzed at the Institute of Marine Research, Bergen, Norway, using a VetScan HM5 (Abaxis, Union City, CA, USA) to measure red blood cell count (RBC), hemoglobin (HGB), hematocrit (HCT), mean corpuscular volume (MCV), mean corpuscular hemoglobin (MCH), mean corpuscular hemoglobin concentration (MCHC), red cell distribution width (RDW), platelet count (PLT), mean platelet volume (MPV), platelet crit (PCT), platelet distribution width (PDW), white blood cell count (WBC), lymphocytes (LYM), monocytes (MON), and neutrophils (NEU).

### 2.3 Liver and plasma fatty acid composition and indexes

The total FA composition in the liver and plasma was analyzed by Mitomega AS using ultrafast gas chromatography (UFGC) (Thermo Electron Corporation, Massachusetts, USA) (17). FA concentrations were expressed as percentages of total FAs by weight (wt%). The Omega-3 Index was defined as the sum of EPA and DHA, expressed as a percentage of the total FA content. The anti-inflammatory index was defined as the ratio of anti-inflammatory FAs (EPA, DHA) to pro-inflammatory FAs (C20:4n-6; arachidonic acid) (18).

The stearoyl-CoA desaturase (SCD) indexes were calculated using the product-substrate ratio, with SCD-18 as oleic acid/stearic acid and SCD-16 as palmitoleic acid/palmitic acid (19). The *de novo* lipogenesis index was calculated according to the following formula: palmitic acid/linoleic acid (19). To assess the degree of unsaturation of the FA pool, the double bond index (DBI) was calculated by summing the concentrations of FAs with one double bond, FAs with two double bonds multiplied by 2, FAs with three double bonds multiplied by 3, FAs with four double bonds multiplied by 4, FAs with five double bonds multiplied by 5, and FAs with six double bonds multiplied by 6, then this sum was divided by the total concentration of FAs.

### 2.4 Glucose tolerance

The animals were fasted for 4 h with free access to water prior to the administration of 2 g/kg glucose by oral gavage. Tail vein blood samples were taken before administration and at 15, 40, 60, and 120 min post-administration, and blood glucose levels were measured using a Contour Next blood glucose meter (Ascensia Diabetes Care, Basel, Switzerland).

### 2.5 Cell culturing

Human satellite cells were isolated from muscle biopsy samples of the *musculus vastus lateralis* (20). The isolation of satellite cells was performed based on the method of Wensaas et al. (21), with modifications described by Pettersen et al. (22). Human skeletal muscle biopsies were obtained after informed written consent and approval by the Regional Committee for Medical and Health Research Ethics South-East, Oslo, Norway (reference number: REK11959). The isolated skeletal muscle cells were cultured and proliferated in DMEM-GlutaMAX (5.5 mM glucose), supplemented with 10% FBS, HEPES (25 mM), gentamicin (50 ng/mL), penicillin (25 IU), streptomycin (25 μg/mL), amphotericin B (1.25 μg/mL), hEGF (10 ng/mL) from Thermo Fisher Scientific (Waltham, MA, US), dexamethasone (0.39 μg/mL), and 0.05% BSA from Sigma-Aldrich (St. Louis, MO, US). Differentiation of myoblasts into myotubes for 7 days was induced at 80–90% confluence by changing the medium to DMEM-GlutaMAX (5.5 mM glucose) supplemented with 2% FBS and 25 pM insulin (Actrapid^®^ Penfill^®^ 100 IE/mL from NovoNordisk (Bagsvaerd, Denmark)).

Human hepatoma cells, Huh7, purchased from ATCC (LGC Standards, Middlesex, UK), were expanded and maintained in Nunc™ Cell and Culture Treated Flasks with DMEM-GlutaMAX (5.5 mmoL/L glucose), supplemented with 10% FBS, HEPES (25 mmoL/L), penicillin (25 IU), streptomycin (25 μg/mL), and amphotericin B (1.25 μg/mL) at 37 °C in a humidified 5% CO_2_ incubator until they reached 80–90% confluence.

### 2.6 Substrate oxidation assay by ^14^CO_2_-trapping in human liver cells and human skeletal cells

To perform the substrate oxidation assays, approximately 12,000 Huh7 liver cells per well and 6,000 myoblasts per well were seeded in a 96-well Corning^®^ CellBIND^®^ tissue culture plate and grown until reaching 70–80% confluence. Myoblasts were further differentiated into myotubes for 7 days. Erucic acid and cetoleic acid were each dissolved in 0.1 M NaOH to a final concentration of 6 mM and subsequently conjugated with 2.4 mM fatty acid-free albumin (BSA) (ratio FA/BSA 2.5/1). The cells were treated with the erucic or cetoleic acid–BSA complexes for 24–48 h prior to the assays. Substrate oxidation assay was assessed by providing a radiolabeled (^14^C) substrate of interest and trapping the released ^14^CO_2_, as described by Wensaas et al. (23). The Huh7 cells and myotubes were given the radiolabeled substrates, either [1-^14^C]oleic acid (0.5 μCi/mL, 100 μM) or D-[^14^C(U)]glucose (0.5 μCi/mL, 200 μM) from PerkinElmer (Boston, MA, US), in DPBS (with MgCl_2_ and CaCl_2_) supplemented with 10 mM HEPES and 10 μM BSA (both from Thermo Fisher Scientific). L-carnitine (1 mM) (Sigma Aldrich) was included in the assay medium for oleic acid oxidation. Respective amounts of the non-radiolabeled substrate were added to obtain the final concentrations of oleic acid. Following trapping, both the produced ^14^CO_2_ and cell-associated (CA) radioactivity were measured using a 2,450 MicroBeta^2^ liquid scintillation counter (PerkinElmer). Protein concentration in each well was determined using the Bio-Rad protein assay kit, allowing the normalization of ^14^CO_2_ and CA data to cellular protein content. Complete substrate oxidation was represented by the measurement of ^14^CO_2_, while uptake was calculated as the sum of ^14^CO_2_ and CA. Safety handling of radioactive materials was performed according to regulations by the Norwegian Radiation and Nuclear Safety Authority (DSA).

### 2.7 Statistical analysis

Statistical analyses were performed using GraphPad Prism^®^ version 10.0. Data were presented as means ± standard deviations (SDs). For the *in vivo* experiment (n = 8 per group), the differences between the control group and the herring oil group receiving the CETO3^®^ supplement were assessed using unpaired two-tailed Student’s *t*-tests assuming equal variances. Unpaired two-tailed Student’s *t*-tests were also used for the *in vitro* experiments on the oxidation of oleic acid and glucose in myotubes (n = 10 for control; n = 5 for erucic acid and cetoleic acid) and Huh7 cells (n = 10 for control; n = 5 for erucic acid and cetoleic acid). A *p*-value of <0.05 was considered statistically significant.

## 3 Results

### 3.1 Mobilization and increase in n-9 and n-11 fatty acids via *de novo* lipogenesis

**Figure 1:**
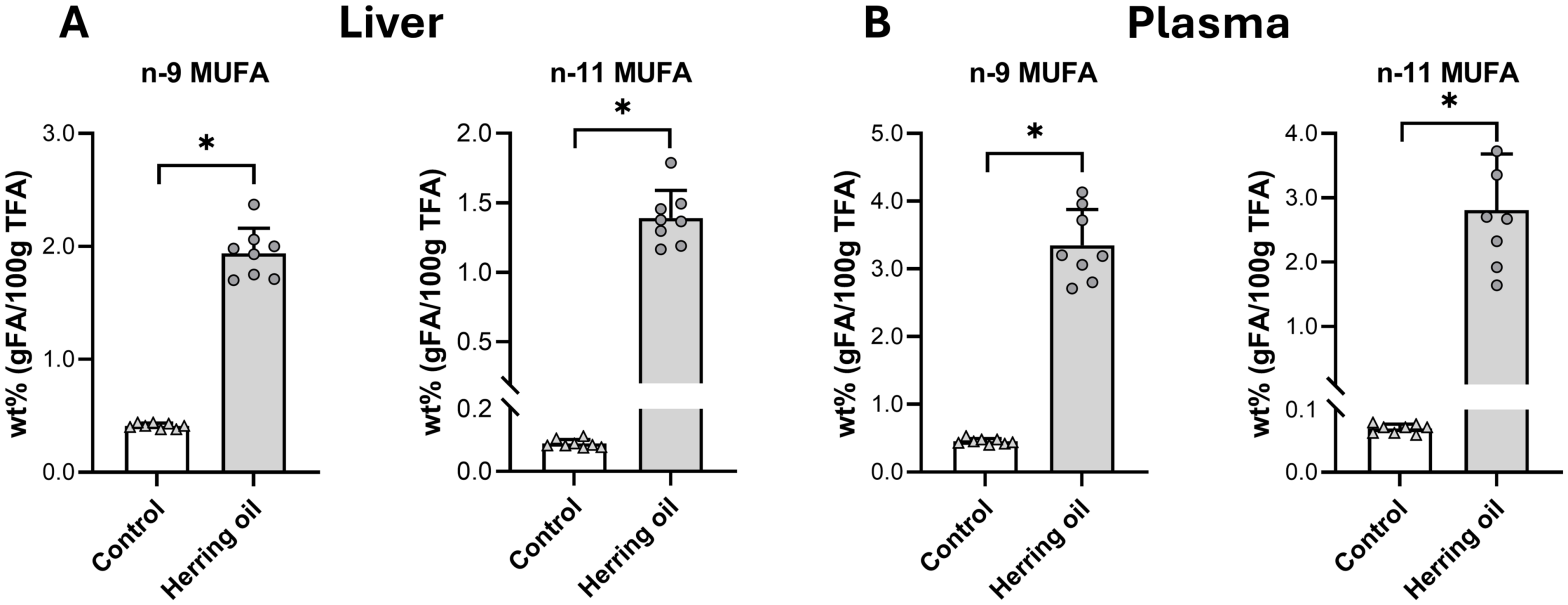
Mobilization of n-9 and n-11 monounsaturated fatty acids (MUFAs) following herring oil intake. The relative proportion (wt%) of n-9 and n-11 MUFAs in the rat liver **(A)** and plasma **(B)**. The control group (n = 8) is represented by white bars, and the herring oil group (n = 8) by grey bars. Values are shown as mean with standard deviation. Statistical significance was determined using an unpaired *t*-test (*p < 0.05, ns = not significant).

**Figure 2:**
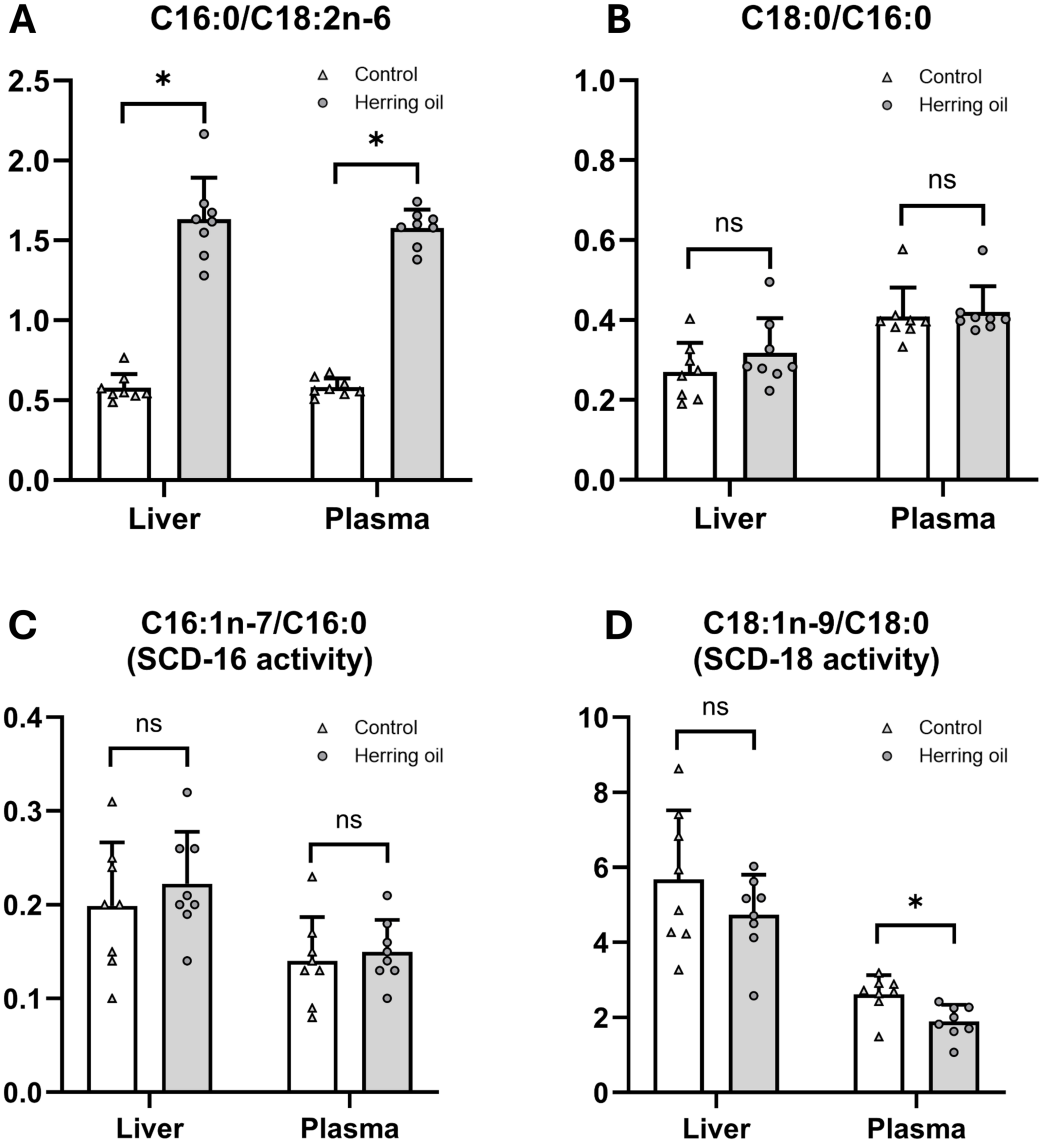
Effect on fatty acid ratios related to *de novo* lipogenesis and desaturase activities after herring oil supplementation in the rats. C16:0/C18:2n-6 ratio **(A)**, C18:0/C16:0 ratio **(B)**, C16:1n-7/C16:0 [SCD-16 activity; **(C)**], and C18:1n-9/C18:0 [SCD-18 activity; **(D)**]. The control group (n = 8) is represented by white bars, while the herring oil group (n = 8) is represented by grey bars. Values are presented as means ± standard deviation. Statistical significance was determined using an unpaired *t*-test (*p < 0.05, ns = not significant).

To assess mechanisms that may contribute to cardiovascular disease risk, we investigated the mobilization of n-9 and n-11 fatty acids and their association with SCD-16 and SCD-18 activity. The consumption of CETO3^®^ changed the FA composition both in the liver and plasma (Supplementary Table 1) and increased the mobilization of n-9 long-chain MUFAs, reflected by C20:1n-9, C22:1n-9, C24:1n-9, and n-11 long-chain MUFAs, such as C20:1n-11 and C22:1n-11, both in the rat liver and plasma (*p* < 0.05; Figures 1A,B). It has been reported that n-11 long-chain MUFAs can only be derived from diets, whereas n-9 long-chain MUFAs can be *de novo* synthesized (24). The changes in the FA (C16:0/C18:2n-6) ratio increased both in the plasma and liver (*p* < 0.05), suggesting that the increased relative proportions of n-9 long-chain MUFAs in plasma are mediated by diet and de novo lipogenesis through the action of FA elongases on C18:1n-9 (Figure 2A). The relative plasma proportion of C18:1n-9 was reduced after the administration of the herring oil (*p* < 0.05; Supplementary Table 1).

It has been reported that long-chain MUFAs can cause transient lipidosis in some organs, which may be influenced by hepatic SCD activity (25). High hepatic SCD-16 activity, indicated by the C16:1n-7/C16:0 ratio, combined with high SCD-18 activity, indicated by the C18:1n-9/C18:0 ratio, in addition to a low C18:0/C16:0 ratio, has been associated with the progression of triglyceride mobilization in the liver (26). Interestingly, the hepatic C16:1n-7/C16:0 and C18:1n-9/C18:0 ratios, serving as proxies for assessing the activities of SCD-16 and SCD-18, and the C18:0/C16:0 ratio were not changed after CETO3^®^ supplementation (*p* > 0.05; Figures 2B–D).

### 3.2 Effect on plasma lipid levels in the rats

**Figure 3:**
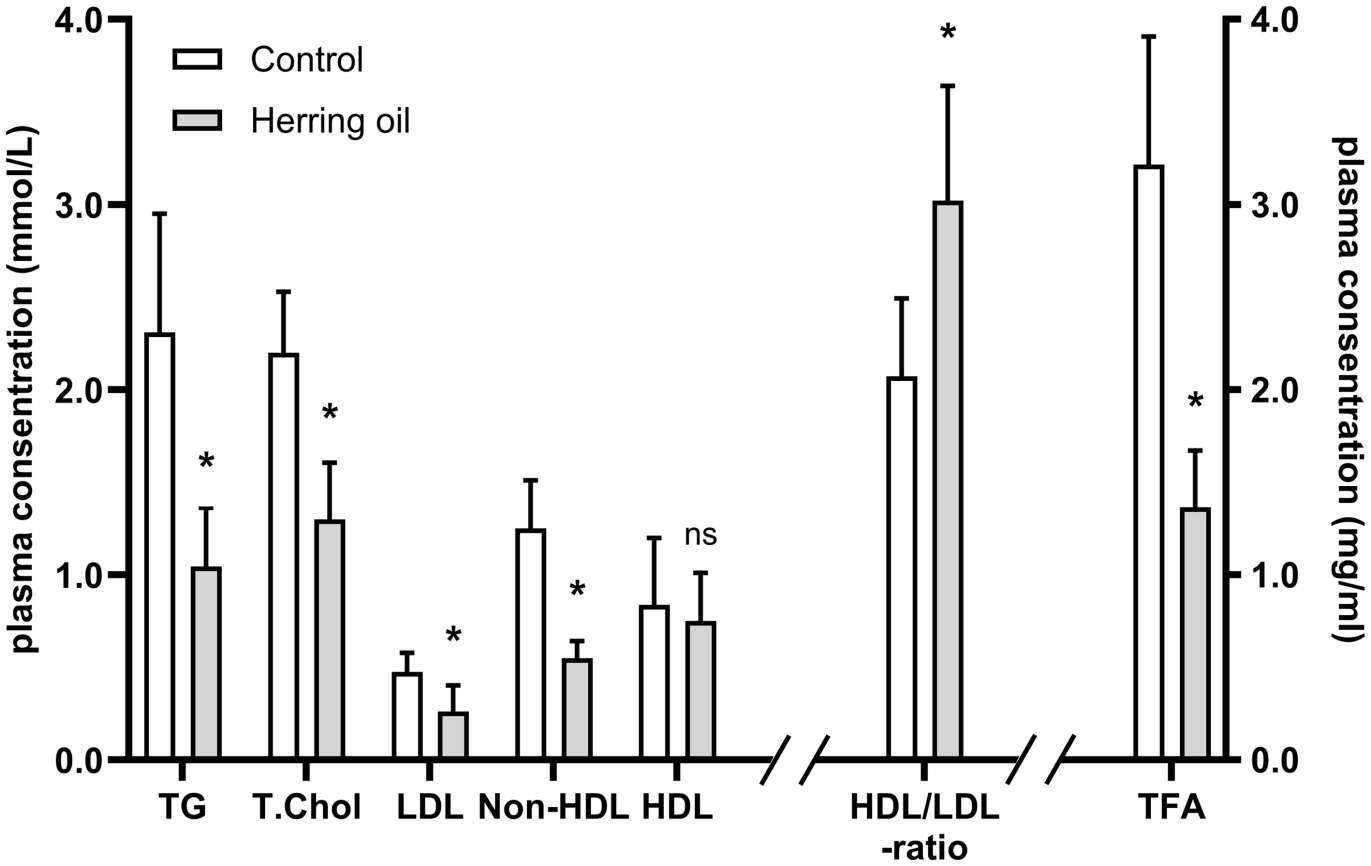
Impact of herring oil supplementation on the plasma lipid profile in the rats. Plasma concentrations (mmol/L) of triglycerides (TG), total cholesterol (T. Chol), LDL cholesterol (LDL), non-HDL cholesterol (non-HDL), and HDL cholesterol (HDL), as well as the HDL/LDL ratio and the plasma concentration (μg/mL) of total fatty acids (TFA). The control group (n = 8) is represented by white bars, and the herring oil group (n = 8) by grey bars. Values are presented as means ± standard deviation. Statistical significance was determined using an unpaired *t*-test (*p < 0.05, ns = not significant).

Increased long-chain MUFAs from herring oil consumption were accompanied by reduced plasma levels of TG (−55%, *p* < 0.05) and total FA (−58%, *p* < 0.05) in the rats (Figure 3). The interference with cholesterol metabolism was confirmed, as the levels of total cholesterol (T. Chol), LDL, and non-HDL cholesterol were reduced by −41, −45%, and −56% (*p* < 0.05), respectively (Figure 3). In contrast, HDL levels were not changed (*p* > 0.05), while a significantly increased HDL/LDL cholesterol ratio (46%, *p* < 0.05) was observed following herring oil supplementation (Figure 3). Hence, CETO3^®^ treatment can reduce plasma lipids in rats.

### 3.3 Safety parameters and glucose tolerance

**Figure 4:**
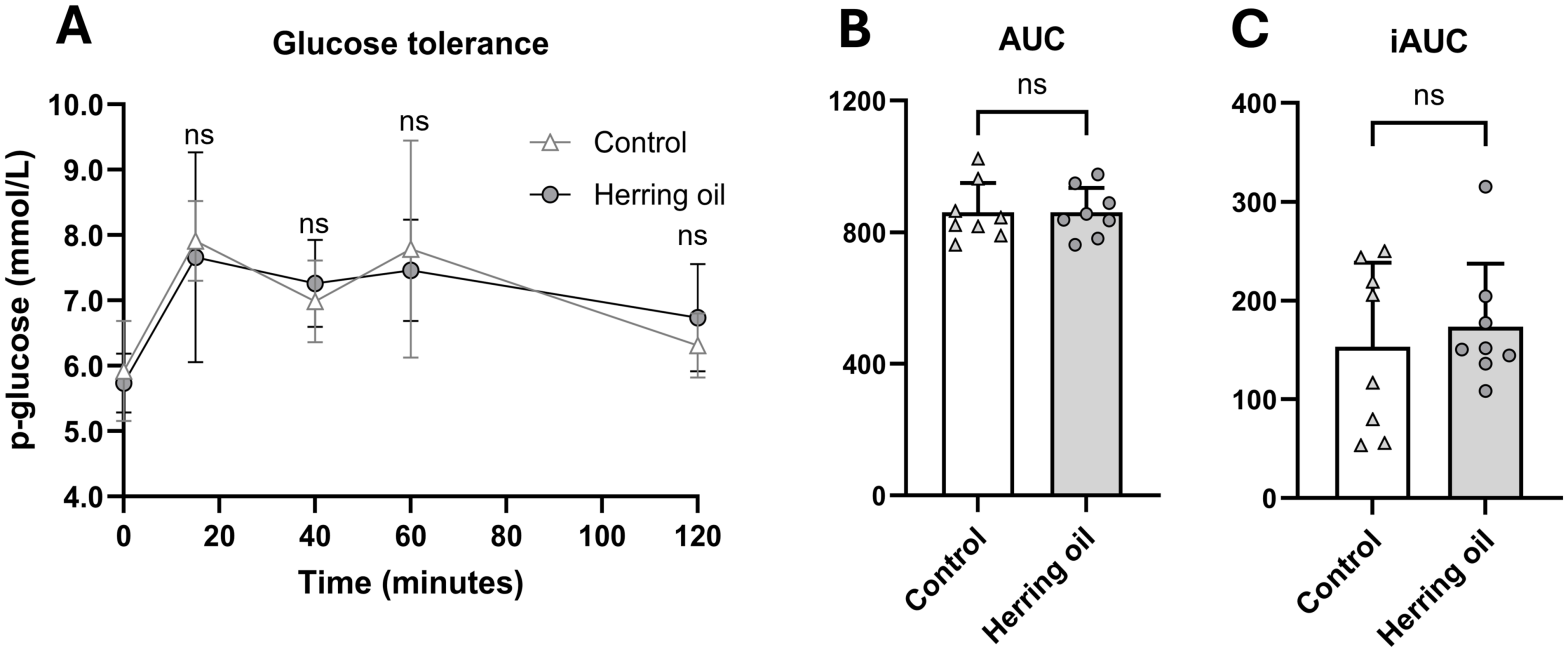
Effect of herring oil supplementation on glucose tolerance in the rats. Glucose tolerance **(A)**, area under the curve [AUC; **(B)**], and incremental area under the curve [iAUC; **(C)**]. The rats were administered 2 g/kg glucose, followed by measurement of blood glucose levels using a Contour Next glucose meter at 15, 40, 60, and 120 min post-administration. The control group (n = 8) is represented by white dots/bars, while the herring oil group (n = 8) is represented by grey dots/bars. Values are presented as means +/- is only showed in A standard deviation. Statistical significance was determined using an unpaired *t*-test (*p < 0.05, ns = not significant).

Weight gain and the masses of fat tissues, liver, brain, heart, kidney, and testis were not significantly changed in the rats following herring oil administration (*p* > 0.05; Supplementary Figure 1). Moreover, plasma safety hematology parameters were not affected by CETO3^®^ treatment, except for an increased red cell distribution width (RDW) observed in the herring oil group (*p* < 0.05; Supplementary Table 3). In addition, glucose tolerance was not affected by herring oil supplementation (*p* > 0.05; Figure 4).

### 3.4 Effect on the oxidation of fatty acids and glucose in the cell models

**Figure 5:**
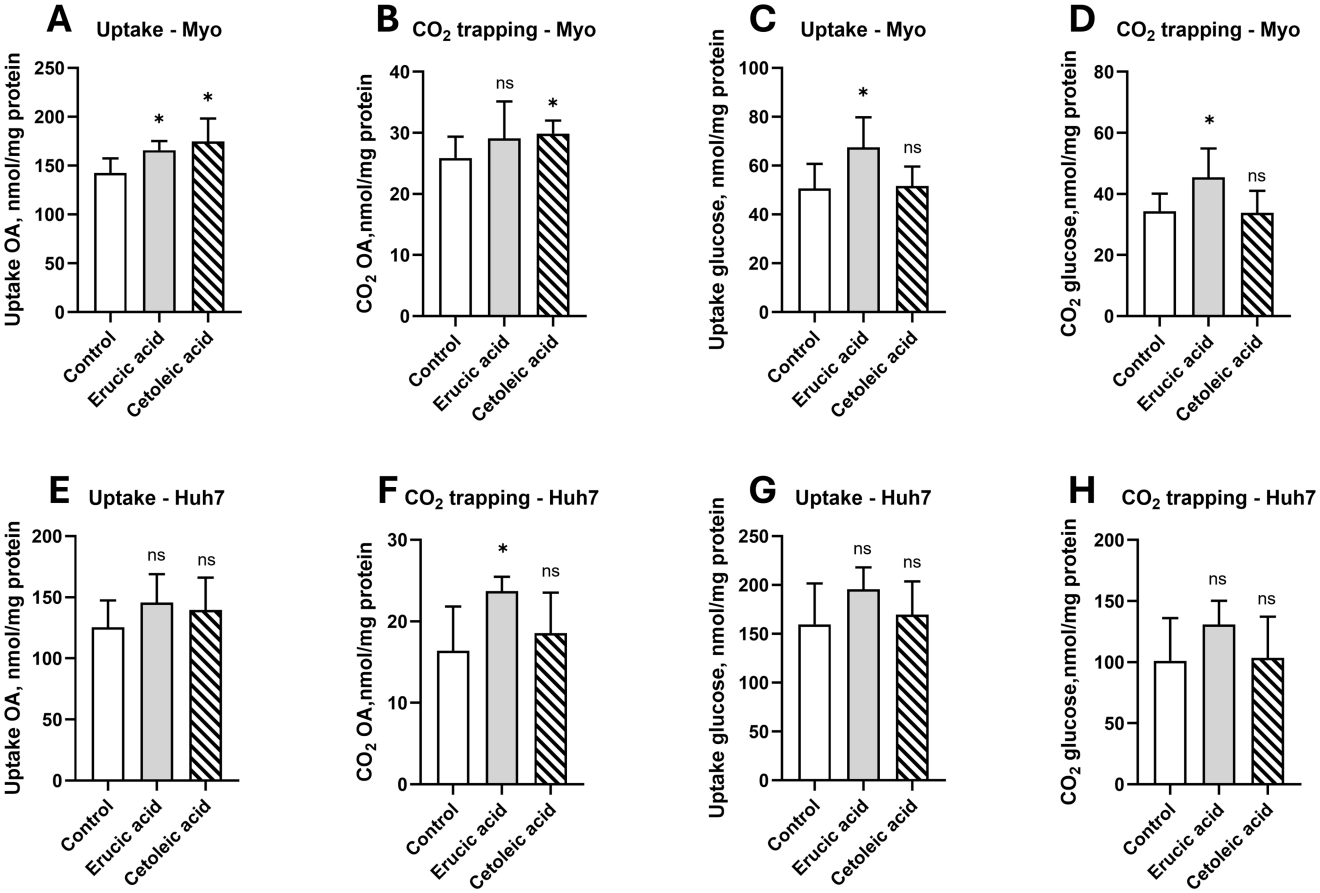
Effect of erucic and cetoleic acid on the metabolism of oleic acid (OA) and glucose in human muscle (Myo) and liver (Huh7) cells. Uptake **(A)** and CO2 trapping **(B)** of OA and uptake **(C)** and CO2 trapping **(D)** of glucose in human myotubes. Uptake **(A)** and CO2 trapping **(B)** of OA and uptake **(C)** and CO2 trapping **(D)** of glucose in human myotubes. Uptake **(E)** and CO2 trapping **(F)** of OA and uptake **(G)** and CO2 trapping **(H)** of glucose in Huh7 cells. For the oxidation of OA, the cultured myotubes and Huh7 cells were treated with 10 μM erucic acid and cetoleic acid in the cell media for 24–48 h before the experiment, except for the uptake of OA in the myotubes, which was treated with 50 μM erucic acid and cetoleic acid. For the oxidation of glucose, the cultured myotubes and Huh7 cells were treated with 100 μM erucic acid and cetoleic acid in the cell media for 24–48 h before the experiment. For the experiment, the cells were treated with either 100 μM [14C] OA or 200 μM [14C] glucose for 4 h. The untreated control group (n = 10) is represented by white bars, while the cells supplemented with erucic acid (n = 5) and cetoleic acid (n = 5) are represented by grey and striped bars, respectively. Values are shown as means +/- standard deviation. Statistical significance was determined using an unpaired *t*-test (*p < 0.05, ns = not significant).

Furthermore, we aimed to investigate whether long-chain MUFAs can affect energy metabolism in cultured human myotubes and liver cells. In the myotubes, we observed increased uptake and oxidation of oleic acid with n-11 FAs (*p* < 0.05), whereas n-9 FAs only increased uptake (*p* < 0.05; Figures 5A,B). Interestingly, n-9 FAs also increased the uptake and oxidation of glucose in the myotubes (*p* < 0.05; Figures 5C,D). However, the uptake and oxidation of both glucose and oleic acid were not changed by n-9 and n-11 FAs in the cultured human liver cells (*p* > 0.05), except for an increased oxidation of OA by n-9 MUFAs (*p* > 0.05; Figures 5E–H).

**Figure 6:**
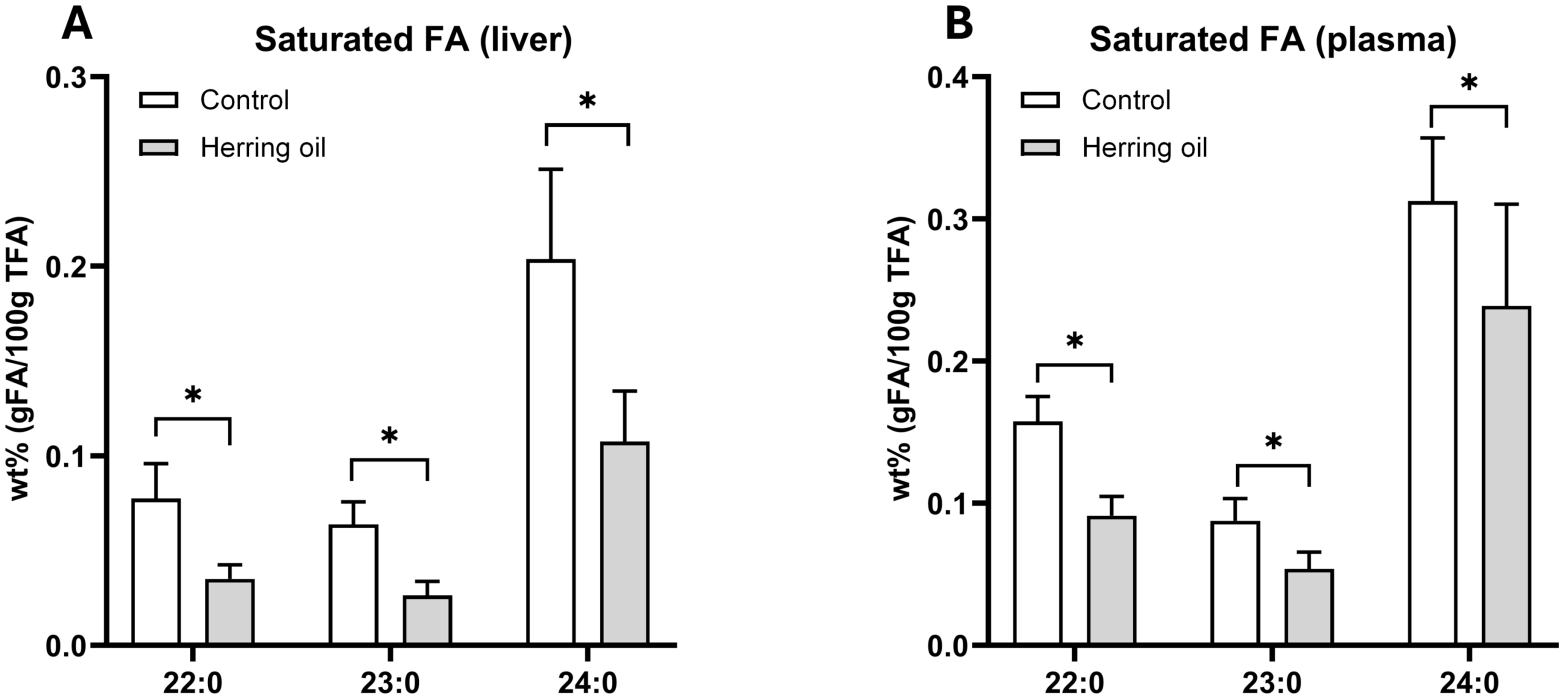
Saturated fatty acid composition in the rats after herring oil intake. The relative proportion (wt%) of saturated fatty acids (FAs) in the liver **(A)** and plasma **(B)**. The control group (n = 8) is represented by white bars, while the herring oil group (n = 8) is represented by grey bars. Values are shown as means +/- standard deviation. Statistical significance was determined using an unpaired *t*-test (*p < 0.05, ns = not significant).

To investigate the effects of long-chain MUFAs on energy metabolism *in vivo*, we utilized a rat model. We found that the levels of long-chain saturated fatty acids (C22:0, C23:0, and C24:0) decreased in both the liver and plasma (*p* < 0.05; Figures 6A,B), which may be due to the increased activity of peroxisomal beta-oxidation following CETO3^®^ supplementation.

### 3.5 Mobilization and changes in n-3 and n-6 fatty acids and anti-inflammation

**Figure 7:**
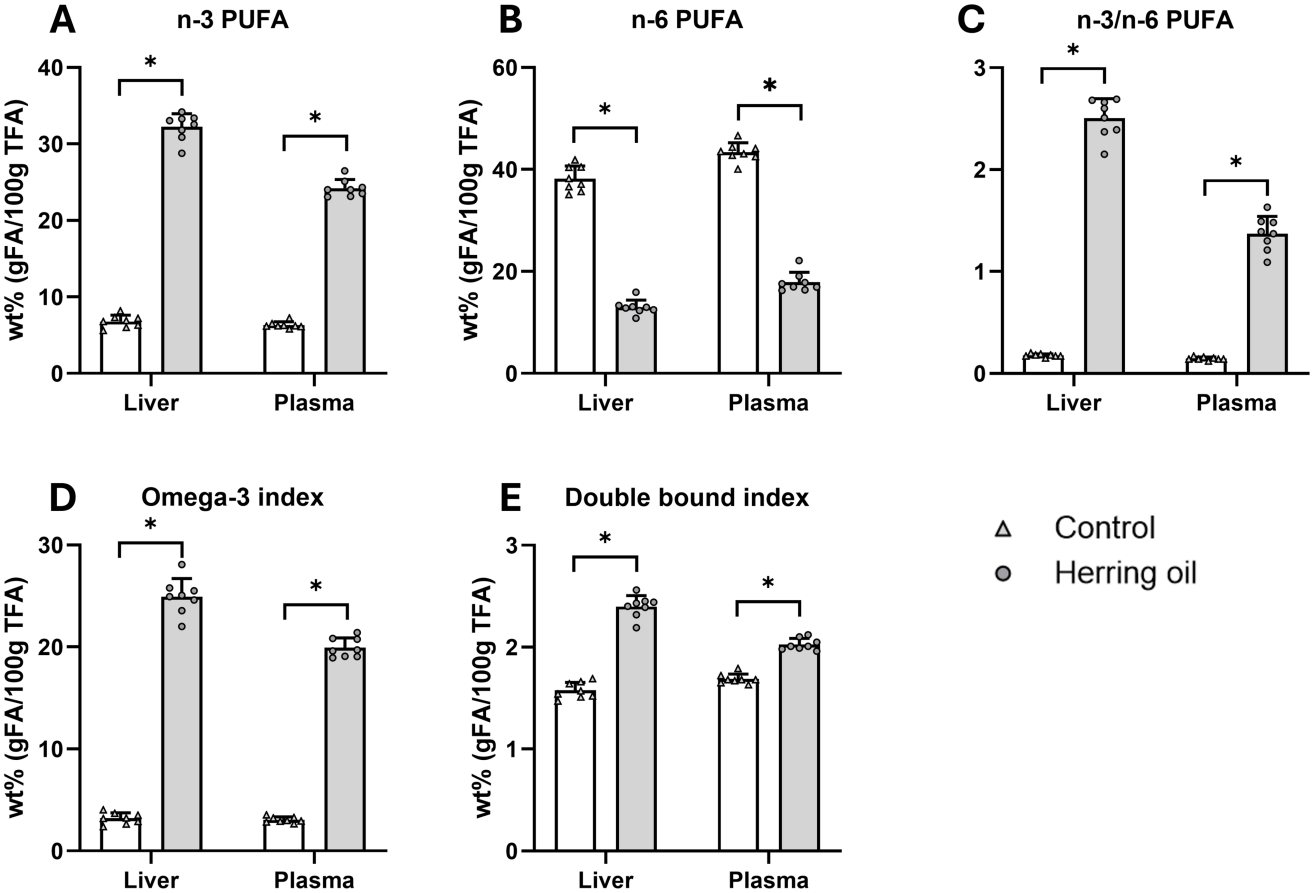
Fatty acid composition in the rat liver and plasma after the consumption of the herring oil. The relative proportion (wt%) of n-3 polyunsaturated fatty acids (PUFAs) **(A)**, n-6 PUFA **(B)**, the n-3/n-6 ratio **(C)**, the Omega-3 Index **(D)**, and the double bond index **(E)**. The control group (n = 8) is represented by white bars, while the herring oil group (n = 8) is represented by grey bars. Values are shown as means +/- standard deviation. Statistical significance was determined using an unpaired *t*-test (*p < 0.05, ns = not significant).

**Figure 8:**
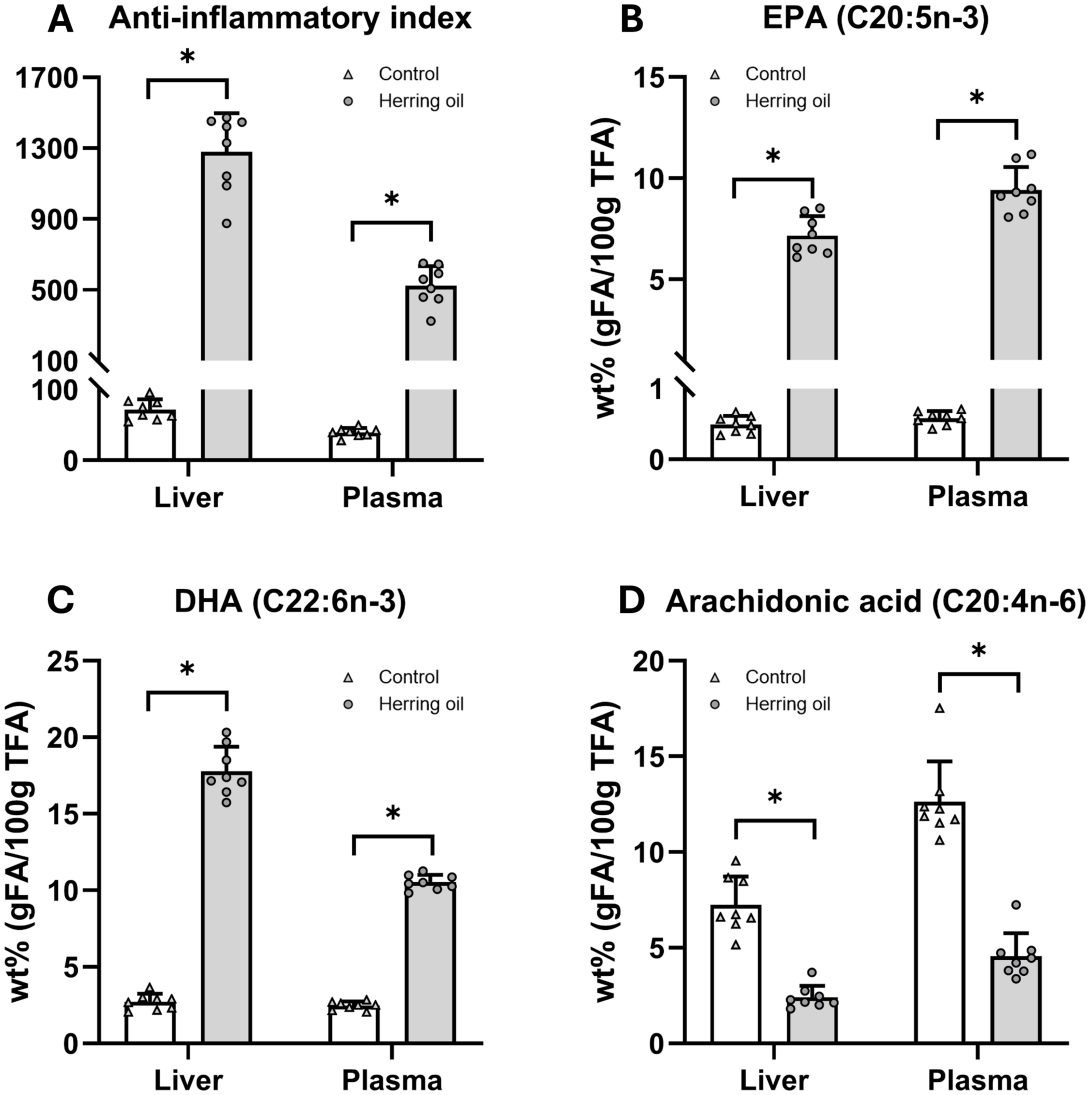
Effect on anti-inflammatory fatty acid indexes after herring oil supplementation in the rat liver and plasma. Anti-inflammatory index **(A)**, and the relative proportion (wt%) of eicosapentaenoic acid (EPA) **(B)**, docosahexaenoic acid (DHA) **(C)**, and arachidonic acid **(D)** in the rat liver and plasma. The control group (n = 8) is represented by white bars, while the herring oil group (n = 8) is represented by grey bars. Values are shown as means +/- standard deviation. Statistical significance was determined using an unpaired *t*-test (*p < 0.05, ns = not significant).

Certain FAs and their ratios are important risk markers related to various diseases within the cardiometabolic syndrome, and reduced hepatic elongation of FA is related to fatty liver (18). In the present study, after herring oil supplementation, changes in hepatic elongases resulted in increased levels of n-3 PUFAs and reduced n-6 PUFAs in both plasma and liver, an increased n-3/n-6 ratio, a higher Omega-3 Index, and an elevated double bond index (*p* < 0.05; Figures 7A–E), accompanied by increased levels of both EPA and DHA (*p* < 0.05; Figures 8B,C). In general, CETO3^®^ treatment increased the relative proportion of EPA and DHA and reduced the relative proportion of n-6 FAs, except for C22:5n-6 (Supplementary Table 1). The anti-inflammatory FA indexes were strongly increased in both the liver and plasma (Figure 8A). This is mostly attributed to the increased proportion of EPA and DHA and the reduced proportion of arachidonic acid (*p* < 0.05; Figures 8B–D).

## 4 Discussion

Overall, we demonstrated that the administration of the CETO3^®^ herring oil in the rats reduced plasma levels of TG, total cholesterol, total fatty acids (TFAs), and LDL and increased the plasma HDL/LDL cholesterol ratio. The observed changes in fatty acid composition may contribute to enhanced anti-inflammatory activity. Furthermore, fatty acid indexes may indicate that both mitochondrial and peroxisomal activities likely play a role in the lipid-lowering effects of CETO3^®^.

Herring oil administration increased the relative proportion of n-9 MUFAs, particularly C20:1n-9, C22:1n-9, and C24:1n-9, as well as n-11 MUFAs, including C20:1n-11 and C22:1n-11, in both the liver and plasma (Figure 1). The observed increase in the C16:0/C18:2n-6 ratio in both plasma and liver (Figure 2) indicates enhanced *de novo* synthesis of FAs, suggesting that the herring oil supplementation may promote an overall shift in the FA profile. Whether this shift is driven directly by dietary intake or by metabolic adaptations should be considered. The increased proportion of n-9 MUFAs in plasma may originate from elongation of de novo synthesized C18:1n-9, which was decreased in plasma after CETO3^®^ administration and the low-fat diet. This is in agreement with previous findings (17, 18).

Previous studies have reported that MUFAs can cause transient lipidosis in some organs (25). While high SCD-16 activity, combined with high SCD-18 activity and a low C18:0/C16:0 ratio, has been associated with the progression of triglyceride mobilization in the liver (26), our study found no alterations in the C18:0/C16:0 ratio or the substrate/product indexes related to SCD-16 and SCD-18 activity after CETO3^®^ herring oil administration (Figure 2). This suggests that the specific herring oil does not impact these metabolic processes related to triglyceride mobilization. Furthermore, the observed decrease in long-chain saturated FAs in both the liver and plasma indicates increased activity of peroxisomal beta-oxidation following herring oil consumption (Figure 6), as peroxisomal beta-oxidation is known to be involved in the reduction of carbon chain length (27). This is consistent with a previous study documenting that administration of fish oil rich in EPA, DHA, and MUFAs stimulates peroxisomal *β*-oxidation, accompanied by enhanced activity of the rate-limiting enzyme ACOX1 (FAO) (28), as well as increased ACOX1 mRNA expression (29).

Whether CETO3^®^ herring oil supplementation also stimulates mitochondrial oxidation should be considered, given that FA oxidation was increased in myotubes after n-3 (20, 21, 30), n-9, and n-11 FAs (Figure 5). In addition, it has been reported that FAs activating mitochondrial function can reduce plasma lipids, including triglycerides, and that this reduction may be partly mediated by increased mitochondrial FA oxidation (18, 31). It has already been documented that an observed increase in *in vitro* fatty acid oxidation is associated with elevated mRNA expression of CPTII, a key enzyme responsible for transporting long-chain fatty acids into the mitochondrial matrix for β-oxidation (29). However, current findings are based on 2D cell models, which have several limitations (32). They do not reflect the complexity of *in vivo* systems, do not adequately mimic the cellular microenvironment, and may not fully represent human physiology as they are not influenced by hormones or other systemic signals.

Indeed, the CETO3^®^ herring oil was shown to lower plasma triglycerides, total cholesterol, LDL cholesterol, and non-HDL cholesterol and to increase the HDL/LDL ratio in the rats (Figure 3). In summary, this enriched monounsaturated herring oil lowered total FAs in plasma, accompanied by FA profile changes in both plasma and liver suggesting a possible increase in anti-inflammatory activity (Figure 8). The FA anti-inflammatory index is related to increased hepatic and plasma content of EPA and DHA and the decrease in arachidonic acid, which was also observed in our study (Figure 8) and has been associated with higher anti-inflammatory activity in humans, thereby potentially reducing inflammatory processes within the vessel wall—a contributing factor in atherosclerosis (5, 6, 33–36). A high n-3/n-6 PUFA ratio has also been shown to influence the pathogenesis of various diseases, including metabolic and cardiovascular disorders and inflammatory conditions (37). Although EPA and DHA have previously been shown to influence FA and plasma lipid profiles, it cannot be excluded that the observed changes in our study are also partly due to the high levels of n-9 and n-11 MUFAs in the herring oil.

In addition, the CETO3^®^ herring oil appears to be safe and well-tolerated as a supplement. The oil did not change organ weight and hematology parameters, except for increased RWD (Supplementary Table 1). Only RDW values outside the normal range are of clinical significance, and no other hematology parameters indicate anemia (38). Previous studies support that the plasma TG-lowering effect and other metabolic benefits of omega-3 FAs observed in rodents are transferable to humans (39). However, further studies in humans are needed to determine the impact of CETO3^®^ supplementation on plasma lipid profiles and to assess the safety and tolerability of high-dose administration.

## 5 Conclusion

In conclusion, we demonstrated that CETO3^®^ herring oil administration to rats reduced risk factors for cardiometabolic syndrome by lowering plasma lipids, including TG and LDL, and total FAs, possibly mediated by peroxisomal and mitochondrial FA oxidation. However, given that the CETO3^®^ herring oil also increased n-3 PUFAs and both hepatic and plasma levels of EPA and DHA, the observed benefits, including potential effects on the FA anti-inflammatory index, may not be solely due to MUFAs of n-11 and n-9 origin.

## Data Availability

The original contributions presented in the study are included in the article/Supplementary material, further inquiries can be directed to the corresponding authors.
