# Supplementary material for: Herring oil rich in long-chain monounsaturated fatty acid (C22: 1n-11) lowers plasma lipids and modulates fatty acid composition, oxidation, and inflammation in rats
Source: Front Nutr. 2025 Sep 26;12:1611166. doi: 10.3389/fnut.2025.1611166 (PMC12513201; doi:10.3389/fnut.2025.1611166)
Supplement: Supplementary file 1 [file Supplementary_file_1.docx]

**Supplementary:**

**E**

**D**

**C**

**B**

**A**

**Supplementary figure 1**: Weight of organs in rats with and without herring oil supplementation. Totalt weight gain (g) for the rats (A), Weight (g) of liver (B), heart (C), kidney (D), and testis (E). The control group (n = 8) is marked with white bars and the herring oil group (n = 8) with grey bars. Values are shown as mean with SD. Unpaired t test was used to determine significance (*p>0.05, ns=).

**Supplementary table** 1: Relative fatty acid composition (wt%) in rat liver for control and herring oil group

| **FA (wt% (g FA/100g TFA))** | **Mean Control (n = 8)** | **Mean Herring oil  (n = 8)** | **p-value (*p<0.05)** |
| --- | --- | --- | --- |
| C16:1n-9 | 0.40 | 0.57 | 0.0038* |
| C16:1n-7 | 3.24 | 3.48 | 0.68 |
| C16:1 | 0.09 | 0.12 | 0.015* |
| C16:0 | 16.31 | 15.48 | 0.19 |
| C17:0 | 0.16 | 0.18 | 0.22 |
| C18:3n-6 | 0.28 | 0.06 | 0.0000* |
| C18:4n-3 | 0.05 | 0.24 | 0.0000* |
| C18:2n-6 | 28.48 | 9.59 | 0.0000* |
| C18:3n-3 | 2.36 | 1.27 | 0.0000* |
| C18:1n-9 | 23.20 | 22.06 | 0.19 |
| C18:1n-7 | 3.82 | 2.17 | 0.0001* |
| C18:1 | 0.16 | 0.25 | 0.0000* |
| C18:0 | 4.41 | 4.85 | 0.44 |
| C20:4n-6 | 7.24 | 2.41 | 0.0000* |
| C20:5n-3 | 0.49 | 7.16 | 0.0000* |
| C20:3n-9 | 0.10 | 0.06 | 0.0000* |
| C20:3n-6 | 0.86 | 0.50 | 0.0000* |
| C20:4n-3 | 0.15 | 0.97 | 0.0000* |
| C20:2n-6 | 0.56 | 0.14 | 0.0000* |
| C20:1n-11 | 0.09 | 0.82 | 0.0000* |
| C20:1n-9 | 0.30 | 1.62 | 0.0000* |
| C20:1n-7 | 0.19 | 0.19 | 0.97 |
| C20:0 | 0.06 | 0.04 | 0.0097* |
| C21:5n-3 | 0.010 | 0.50 | 0.0000* |
| C22:5n-6 | 0.18 | 0.18 | 0.91 |
| C22:6n-3 | 2.71 | 17.79 | 0.0000* |
| C22:4n-6 | 0.57 | 0.07 | 0.0000* |
| C22:5n-3 | 1.03 | 4.30 | 0.0000* |
| C22:2n-6 | 0.016 | 0.005 | 0.0000* |
| C22:1n-11 | 0.002 | 0.57 | 0.0000* |
| C22:1n-9 | 0.04 | 0.09 | 0.0001* |
| C22:1n-7 | 0.02 | 0.02 | 0.27 |
| C22:0 | 0.08 | 0.03 | 0.0000* |
| C23:0 | 0.06 | 0.03 | 0.0000* |
| C24:1n-9 | 0.07 | 0.23 | 0.0000* |
| C24:0 | 0.20 | 0.11 | 0.0002* |

**Supplementary table 2:** Relative fatty acid composition (wt%) in rat plasma for control and herring oil group

| **FA (wt% (g FA/100g TFA))** | **Mean Control (n = 8)** | **Mean Herring oil (n = 8)** | **p-value (*p<0.05)** |
| --- | --- | --- | --- |
| C16:1n-9 | 0.28 | 0.43 | 0.0002* |
| C16:1n-7 | 2.31 | 2.81 | 0.18 |
| C16:1 | 0.07 | 0.14 | 0.0000* |
| C16:0 | 16.49 | 19.07 | 0.0002* |
| C17:0 | 0.21 | 0.26 | 0.0003* |
| C18:3n-6 | 0.22 | 0.09 | 0.0000* |
| C18:4n-3 | 0.03 | 0.47 | 0.0000* |
| C18:2n-6 | 28.47 | 12.10 | 0.0000* |
| C18:3n-3 | 2.13 | 0.80 | 0.0000* |
| C18:1n-9 | 17.15 | 14.71 | 0.0069* |
| C18:1n-7 | 3.18 | 2.14 | 0.0003* |
| C18:1 | 0.24 | 0.40 | 0.0000* |
| C18:0 | 6.73 | 8.02 | 0.056 |
| C20:4n-6 | 12.63 | 4.56 | 0.0000* |
| C20:5n-3 | 0.58 | 9.40 | 0.0000* |
| C20:3n-9 | 0.11 | 0.08 | 0.0001* |
| C20:3n-6 | 0.87 | 0.67 | 0.0005* |
| C20:4n-3 | 0.14 | 0.60 | 0.0000* |
| C20:2n-6 | 0.51 | 0.22 | 0.0000* |
| C20:1n-11 | 0.07 | 0.59 | 0.0000* |
| C20:1n-9 | 0.25 | 2.34 | 0.0000* |
| C20:1n-7 | 0.40 | 0.43 | 0.62 |
| C20:0 | 0.12 | 0.10 | 0.0016* |
| C21:5n-3 | 0.005 | 0.23 | 0.0000* |
| C22:5n-6 | 0.16 | 0.14 | 0.49 |
| C22:6n-3 | 2.47 | 10.52 | 0.0000* |
| C22:4n-6 | 0.49 | 0.08 | 0.0000* |
| C22:5n-3 | 0.97 | 2.19 | 0.0000* |
| C22:2n-6 | 0.03 | 0.03 | 0.24 |
| C22:1n-11 | 0.003 | 2.22 | 0.0000* |
| C22:1n-9 | 0.04 | 0.18 | 0.0000* |
| C22:1n-7 | 0.07 | 0.08 | 0.058 |
| C22:0 | 0.16 | 0.09 | 0.0000* |
| C23:0 | 0.09 | 0.05 | 0.0003* |
| C24:1n-9 | 0.16 | 0.82 | 0.0000* |
| C24:0 | 0.31 | 0.24 | 0.024* |

**Supplementary table 3**: Safety hematology parameters for rats with and without herring oil supplementation

| **Analyte** | **Mean Control (n = 8)** | **Mean**  **Herring oil (n = 8)** | **p-value (*p<0.05)** |
| --- | --- | --- | --- |
| WBC 10^9/l | 10.41 | 9.62 | 0.53 |
| LYM 10^9/l | 7.93 | 7.20 | 0.52 |
| MON 10^9/l | 0.51 | 0.52 | 0.89 |
| NEU 10^9/l | 1.98 | 1.90 | 0.72 |
| RBC 10^12/l | 9.29 | 9.19 | 0.61 |
| HGB g/dl | 14.71 | 14.69 | 0.95 |
| HCT % | 45.54 | 46.12 | 0.64 |
| MCV fl | 49.00 | 50.13 | 0.41 |
| MCH pg | 15.86 | 15.99 | 0.76 |
| MCHC g/dl | 32.33 | 31.88 | 0.37 |
| RDWs fl | 33.49 | 37.70 | 0.0001* |
| RDWc % | 18.36 | 20.00 | 0.0004* |
| PLT 10^9/l | 608.6 | 517.3 | 0.16 |
| PCT % | 0.44 | 0.38 | 0.13 |
| MPV fl | 7.33 | 7.21 | 0.67 |
| PDWs fl | 8.75 | 8.86 | 0.80 |
| PDWc % | 33.76 | 34.18 | 0.50 |
